# Phenolic content variability and its chromosome location in tritordeum

**DOI:** 10.3389/fpls.2014.00010

**Published:** 2014-01-30

**Authors:** José F. Navas-Lopez, Francisco J. Ostos-Garrido, Almudena Castillo, Antonio Martín, Maria J. Gimenez, Fernando Pistón

**Affiliations:** Departamento de Mejora Genética Vegetal, Instituto de Agricultura Sostenible - Consejo Superior de Investigaciones CientíficasCórdoba, Spain

**Keywords:** plant breeding, antioxidant, healthy, variability, nutritive, chromosome sustitution, wheat, flour quality

## Abstract

For humans, wheat is the most important source of calories, but it is also a source of antioxidant compounds that are involved in the prevention of chronic disease. Among the antioxidant compounds, phenolic acids have great potential to improve human health. In this paper we evaluate the effect of environmental and genetic factors on the phenolics content in the grain of a collection of tritordeums with different cytoplasm and chromosome substitutions. To this purpose, tritordeum flour was used for extraction of the free, conjugates and bound phenolic compounds. These phenolic compounds were identified and quantified by RP-HPLC and the results were analyzed by univariate and multivariate methods. This is the first study that describes the composition of phenolic acids of the amphiploid tritordeum. As in wheat, the predominant phenolic compound is ferulic acid. In tritordeum there is great variability for the content of phenolic compounds and the main factor which determines its content is the genotype followed by the environment, in this case included in the year factor. Phenolic acid content is associated with the substitution of chromosome DS1D(1H^ch^) and DS2D(2H^ch^), and the translocation 1RS/1BL in tritordeum. The results show that there is high potential for further improving the quality and quantity of phenolics in tritordeum because this amphiploid shows high variability for the content of phenolic compounds.

## 1. Introduction

Cereals, particularly wheat, are the most important food source for human. Numerous studies have demonstrated the key role of whole grains in human health benefits, since they are known to be protective against chronic diseases. These data provide further support for recommendations to increase consumption of whole grains and its use as healthy products (Marquardt et al., 2004; Flight and Clifton, 2006; de Munter et al., 2007; Jonnalagadda et al., 2011). A wide range of phytochemicals with antioxidant activity present in cereal grains has been indicated to be responsible for these health protection qualities. These compounds belong to chemical groups such as polyphenols, carotenoids, and plant sterols. Among the different antioxidants present in wheat, phenolic compounds seem to have the greatest potential of being beneficial to health (Beta et al., 2005; Mpofu et al., 2006).

Phenolic acids are the most frequent phenolic compounds in cereals; these can be found as free, bound and conjugated forms. Most phenolic acids are bound by ester-linked to the cell wall polymers (Irakli et al., 2012). The main phenolic acids in wheat are ferulic and ρ-coumaric acids, both associated with cell-wall constituents (Zhou et al., 2004, 2005; Okarter et al., 2010). It has been described that factors such as the genotype, environmental factors and genotype-environment interactions can influence total phenolic compounds content and the relative content of different phenolics fractions. Yu and Zhou (2005) described the effect of the location, on the content of phenolic compounds; Moore et al. (2006) observed significant correlations between temperature stress or solar radiation and some antioxidant contents. Furthermore, Fernandez-Orozco et al. (2010) showed that environmental factors did not equally affect to all phenolic compounds fractions, the fractions of free phenolic compounds and conjugates being more affected than the fraction of bound phenolics.

In addition, the content of phenolic compounds is affected by the genotype and the genotype x environment interaction. The results of Mpofu et al. (2006) showed that environmental effects on the content of phenolic compounds were considerably larger than the genotype effect, and genotype × environment interaction was small for all parameters as compared to both main factors. The study of Fernandez-Orozco et al. (2010) also showed that the environmental effects were larger than genotypic differences, especially for the low abundance of free and conjugated phenolic acid fractions. Li et al. (2008) reported differences in phenolic content between varieties, and they suggested that it may be possible to develop varieties with a high content of phenolic acids. The results of Shewry et al. (2010) corroborated previous reports and demonstrated that heritable variation in the content of bioactive components can be exploited by breeders to develop new cultivars with enhanced health benefits. Triticeae wild species might also be a valuable source of variability, but transferring this variability to cultivated species presents multiple problems. That is not the case of the wild species *Hordeum chilense* Roem. et Schultz. whose variability for traits of interest may be used in both durum and bread wheat breeding using the amphiploids called tritordeums (×Tritordeum Ascherson et Graebner) (Martin and Sanchez-Mongelaguna, 1982; Martín et al., 1998, 1999).

Moreover, other qualities of tritordeum confer on it the potential to be part of a healthy diet and it has recently been introduced to the market as an alternative food cereal (www.tritordeum.com). For example, tritordeum has a high content of carotenoids, such as lutein, which has been associated with prevention against different diseases (Atienza et al., 2007; Mellado-Ortega and Hornero-Méndez, 2012). To the present, little is known about the content and variability of phenolic compounds, especially phenolic acids of tritordeum. In addition, factors affecting the content of phenolic compounds have not been determined and it is not known whether they are the same as those involved in the content of phenolic compounds in wheat. Furthermore, little is known about the genetic and chromosomal location of the trait of high/low content of phenolic compounds. In order to carry out an efficient improvement of this character to increase health benefits, more information on the factors affecting phenolic content needs to be collected.

The aim of the present work was to evaluate the whole grain phenolic acids content of accessions of hexaploid tritordeum and to assess the effect of the environment and some genetic factors such as the presence of barley or wheat cytoplasm; substitution of chromosomes 1H^ch^, 2H^ch^, and 5H^ch^; and the 1RS/1BL translocation, on phenolic acid content.

## 2. Materials and methods

### 2.1. Plant material

Thirty seven accessions of hexaploid tritordeum originating from different stages of the tritordeum breeding program were evaluated (Table 1). The previous genotyping of the tritordeum lines (Castillo et al., 2013) allowed their characterization. Different groups were identified according their genomic constitution (1) cytoplasm, (2) chromosome substitutions, and (3) 1RS/1BL translocation. Tritordeum grains were ground using a cyclonic mill (Ciclotec lab mill, Tecator) with 1 mm mesh to produce wholemeal flour. Samples were stored at 4°C until analysis.

**Table 1:** **Tritordeum collection used in this study**.

**Line**	**Year**	**Cytoplasm**	**1RS/1BL**	**Substitutions**
223	10;11;12	*H. chilense*	N	N
294	10;11;12	Wheat	N	DS1D(1H^ch^)
295	10;11;12	*H. chilense*	N	N
296	10;11;12	Wheat	N	N
320	10;11;12	*H. chilense*	N	N
322	10;11;12	*H. chilense*	N	N
323	10;11;12	*H. chilense*	N	N
324	10;11;12	*H. chilense*	N	N
325	10;11;12	*H. chilense*	N	N
326	10;11;12	*H. chilense*	N	N
327	10;11;12	*H. chilense*	N	N
328	10;11;12	Wheat	N	DS1D(1H^ch^)
400	10;11;12	Wheat	N	DS2D(2H^ch^)
409	10;11;12	Wheat	R	DS5D(5H^ch^)
410	10;11;12	*H. chilense*	R	N
411	10;11;12	*H. chilense*	R	N
412	10;11;12	*H. chilense*	N	N
413	10;11;12	*H. chilense*	R	N
414	10;11;12	Wheat	R	N
415	10;11;12	Wheat	R	DS2D(2H^ch^)
416	10;11;12	Wheat	R	DS2D(2H^ch^)
417	10;11;12	*H. chilense*	R	DS2D(2H^ch^)
418	10;11;12	NA	N	N
419	10;11;12	Wheat	N	N
421	10;11;12	Wheat	R	DS2D(2H^ch^);DS5D(5H^ch^)
422	10;11;12	Wheat	N	N
423	10;11;12	*H. chilense*	N	N
424	10;11;12	Wheat	R	DS2D(2H^ch^);DS5D(5H^ch^)
425	10;11;12	Wheat	R	DS2D(2H^ch^);DS5D(5H^ch^)
427	10;11;12	Wheat	N	N
429	10;11;12	Wheat	R	N
430	10;11;12	*H. chilense*	R	DS5D(5H^ch^)
431	10;11;12	*H. chilense*	N	DS5D(5H^ch^)
432	10;11;12	*H. chilense*	R	N
436	10;11;12	*H. chilense*	N	DS5D(5H^ch^)
621	10;11;12	NA	NA	NA
631	10;11;12	Wheat	N	N

10;11;12, years 2010, 2011 and 2012 respectively; N, no translocation or no substitutions; R, 1RS/1BL translocation; NA, no available.

### 2.2. Phenolic compounds extraction

#### 2.2.1. Extraction of free phenolics

Free phenolics (and also bound and conjugate phenolics) were extracted using the method previously reported by Okarter et al. (2010) with some modifications. One hundred milligram of wholemeal flour was blended with 670 μl of 80% chilled acetone in 2 mL tube. Inside of the tube we added a 5 mm stainless steel ball to facilitate the mixing in a Retsch MM200 ball mill for 1 min at 60% of power. The mixture was centrifuged at 2500 ×g for 10 min The supernatant was removed and the remaining pellet was extracted two times more with 80% chilled acetone following the same process. The supernatants were pooled and evaporated in a Speed Vac vacuum centrifuge (Savant) to dryness. The solution was then reconstituted with methanol/hydrochloric acid (1 M; 85:15, v/v), filtered through a 0.45 μm filter, and stored at −20°C until analysis.

#### 2.2.2. Extraction of bound phenolics

Insoluble-bound phenolics were extracted from the residue after the free phenolics extraction. The residue was then washed three times with distilled water to remove the water soluble sugars which may gelate and hinder the extraction of bound phenolics. After washing, the residual pellet was digested with 1.5 ml of a 2N sodium hydroxide solution. The mixture was blended in a Retsch MM200 ball mill for 1 min at 60% of power and incubated for 1 h at room temperature under gentle shaking in darkness. Both the sodium hydroxide and the mixture of sodium hydroxide and the pellet were degassed with nitrogen. After digestion, the tubes were centrifuged for 2 min at 8000 ×g and each supernatant was transferred to a new tube. The digestion was stopped with fumant chloride acid (75%) to reach a pH 2. We extracted the phenolics by adding 670 μl of ethyl acetate, mixing by vortex for 1 min and centrifuging for 10 min at 7000 × g. The supernatant was transferred to a clean tube. This process was repeated three times and the supernatants pooled. The ethyl acetate was evaporated in a Speed Vac vacuum centrifuge (Savant). The solution was then reconstituted with mehtanol/hydrochloric acid (1 M; 85:15, v/v), filtered through a 0.45 μm filter, and stored at −20°C until analysis.

#### 2.2.3. Extraction of soluble-conjugated phenolic compounds

Soluble-conjugated phenolics were extracted from half volume of free phenolics extracts using the method reported above for the bound phenolics with some minor modifications. To the residue from evaporation of the acetone of half volume of free phenolics extracts, we added 1.5 ml of 2N sodium hydroxide and from this step we followed the same process like in the extraction of bound phenolics.

### 2.3. HPLC method

The phenolics extracts (100 μl) were applied to a Eclipse XDB-C18 reverse phase analytical column (4.6 × 150 mm, 5 μm particle size; Agilent Technologies) using a 1200 Series Quaternary LC System liquid chromatograph (Agilent Technologies) with a DAD UV-V detector. We eluted the phenolic compounds using as solvent A a mix of water and 0.01% trifluoroacetic (TFA), and as solvent B a mix of acetonitrile (ACN) and 0.01% of TFA, according to the following method: 1 ml min^−1^, and a gradient from 7% to 16% of solvent B (to 100% with solvent A) over 22.5 min followed by a 65% of solvent B over 5 min to wash the column. Absorbance was monitored with the DAD UV-V module at 280 nm.

### 2.4. Experimental design and statistical analysis

All analyses were conducted with the statistical software R version 2.12.1 (R Development Core Team, 2012). The experimental design was a completely randomized design replicated for 3 years (2010–2012) with a 2 m^2^ plots. Data were adjusted to a lineal model with the function *lm* and factors effects were checked by a analysis of variance with the function *anova*. The lineal models were adjusted with the factors “Year,” “Line,” “Substitution,” “1RS/1BL” and “Cytoplasm” (see models formula in section 3). Factor “Year” has three levels (years 2010, 2011, and 2012), factor “Line” has 37 (see Table 3), factor “Substitution” has five levels (DS1D(1H^ch^), DS2D(2H^ch^), DS5D(5H^ch^), DS2D(2H^ch^) DS5D(5H^ch^) and N), “1RS/1BL” has two levels (R (1RS/BL translocation) and N (no translocation)) and “Cytoplasm” has two levels (cytoplasm from *H. chilense* and from Wheat).

The normality and heteroscedasticity assumptions were tested by plotting the residuals versus the predicted values and Q-Q plots. In the cases where the conditions of data normality and homogeneity of variances were violated the Box-Cox transformation was applied (function *powerTransform*, package *car*) (Fox and Weisberg, 2011). The differences between tritordeum lines were assessed using *post hoc* multiple-comparison test (function *glht*, package *multcomp*) (Hothorn et al., 2008). The method used for multiple-comparison was a Tukey contrast with a *p-values* adjustment type *free* (see package *multcomp* for more information).

To identify the relative importance of each factor in the total set of data, we performed a multivariate analysis of variance (MANOVA) with the function *lm*. The multivariate models were adjusted with the factors “Year,” “Line,” “Substitution,” “1RS/1BL” and “Cytoplasm” (see models formula in section 3). As an exploratory graphic, we generated a Principal Components Analysis (PCA) to demonstrate trends of tritordeum lines in relation to the variables studied using the function *PCA* (package *FactoMineR*) (Husson et al., 2013). The package *maptools* was used to avoid the label overlapping in the ordination plot (Lewin-Koh et al., 2012).

## 3. Results

### 3.1. Identification of phenolic compounds

**Figure 1:**
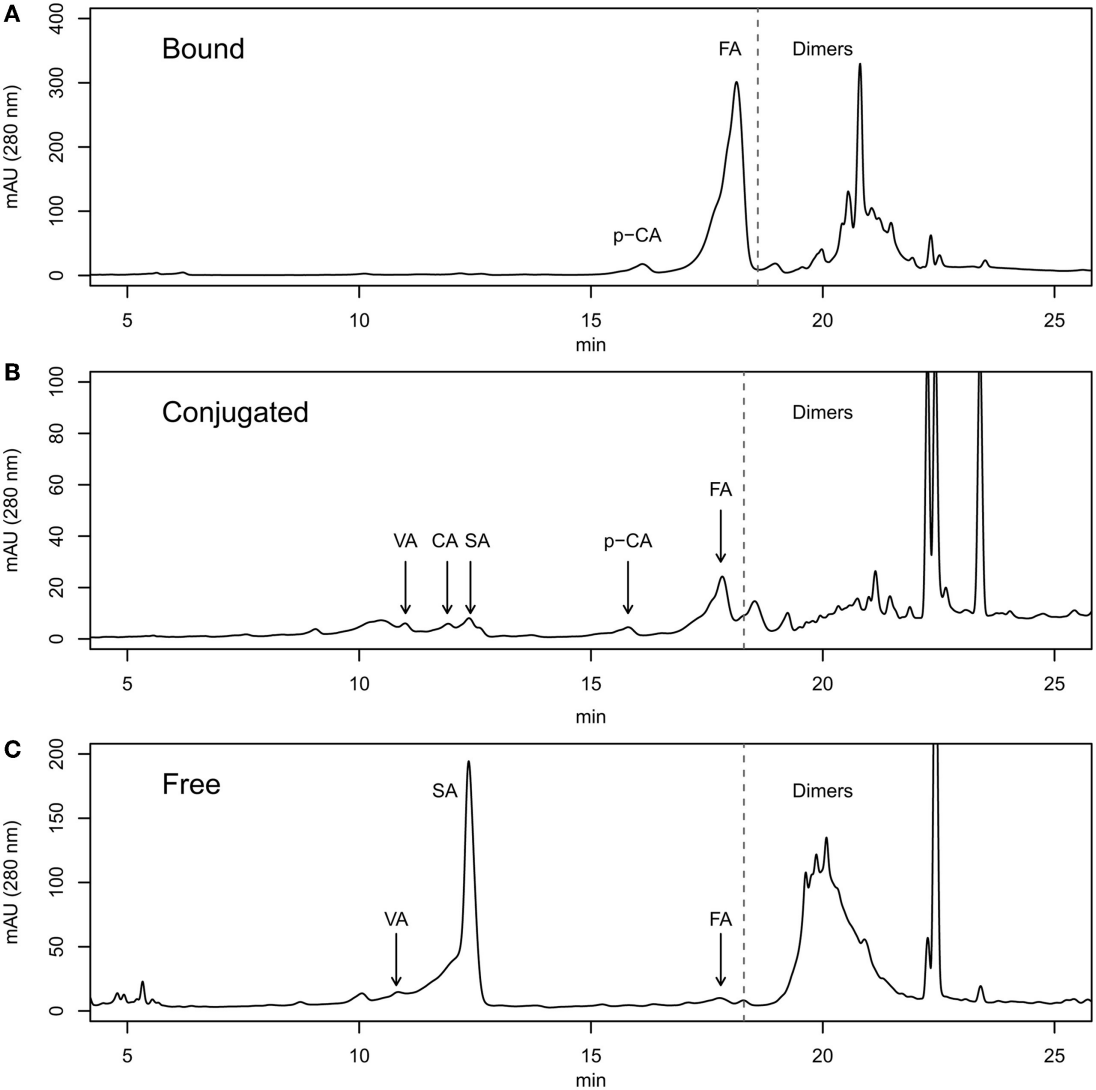
**RP-HPLC chromatograms of the bound **(A)**, conjugated **(B)**, and free **(C)** phenolics fractions from tritordeum grain**.

For identification of phenolic compounds by RP-HPLC we used pure compounds from SIGMA-ALDRICH (http://www.sigmaaldrich.com/). The pure compounds ferulic acid (FA, cat# 46278), ρ-coumaric acid (pCA, cat# C9008), caffeic acid(CA, cat# C0625), vanillic acid (VA, cat# 94770) and syringic acid (SA, cat# S6881) were dissolved in methanol at a concentration of 1 mg/ml and injected in HPLC according to the program described in section 2. We used the chromatograms of pure compounds for their identification in the grain samples analyzed (Figure S1). For the identification, we used mainly the retention times (ferulic acid ≈17 min; ρ-coumaric acid ≈15.2 min; caffeic acid ≈11.2 min; vanillic acid ≈10.4 min.; syringic acid ≈11.7 min). In Figure S1A we showed the chromatogram of a mix of the same amount of each phenolic compound. The area and height at 280 nm of the monomeric peaks were very similar. This means that the response factor (absorbance by weight unit) was very similar for all pure phenolic compounds studied in this paper. The peaks appearing from minute 19 onwards were considered dimeric phenolics (phenolic compounds all together). We tried to identify the phenolic compounds peaks in the grain samples using retention time, but we found small variations in the compounds peak migrations times and also small unknown peaks near phenolic compounds peaks. In these cases, we used the spectrum of the pure compounds from 210 to 400 nm and we compare it with the peaks present in the samples (Figure S2) using the tool for detection of pure spectra of the ChemStation software. When the peak spectrum showed no coincidence above 80% with any of the pure spectra no peak was identified. Moreover, we did not associate the peaks of the dimeric region with any particular phenolic compound and we could not measure the total amount of each individual phenolic compound. We extracted free, bound and soluble-conjugated phenolic compounds from whole flour of a set of Tritordeum lines assayed during three consecutive years. The different fractions of phenolic compounds were injected into liquid chromatograph columns as described in section 2 (Figure 1). In Figure 1A we plot a typical chromatogram of the bound phenolic fraction. This chromatogram presented pCA, FA and dimers. In the fraction of soluble-conjugated phenolics (Figure 1B) we found all phenolic compounds studied in this work (FA, pCA, CA, VA, and SA), although some of them at very low amounts. Finally, the fraction of free phenolics VA, SA, FA, and dimers is presented (Figure 1C).

### 3.2. Quantification of phenolic acid composition

The relative quantification is shown in Table 2. The values shown are the mean percentages and standard deviation of all lines and years. The most abundant compound in the fraction of bound phenolics is ferulic acid followed by ρ-coumaric. Ferulic acid is also the main compound in the fraction of conjugated phenolics. In the fraction of free phenolics is more abundant syringic acid. In general, the main compound quantitatively is the ferulic acid and the second most abundant is the syringic acid. With respect to the fractions, the highest content of phenolic compounds are in the bound fraction and the lowest in the conjugated.

**Table 2:** **Table with the mean percentage and standard deviation of each phenolic acid relative to the total phenolic compounds**.

**Phenolic compound**	**Mean (%)**	***SD***
Bound ferulic acid	68.90	6.50
Bound vanillic acid	0.00	0.00
Bound ρ-coumaric acid	3.50	1.10
Bound syringic acid	0.10	0.10
Bound caffeic acid	0.10	0.10
Bound dimers	6.70	0.80
Free ferulic acid	1.30	1.00
Free vanillic acid	0.80	0.40
Free ρ-coumaric acid	0.30	1.10
Free syringic acid	15.60	6.90
Free caffeic acid	0.40	1.60
Free dimers	7.60	1.50
Conjugated ferulic acid	6.20	2.10
Conjugated vanillic acid	0.70	0.20
Conjugated ρ-coumaric acid	0.70	0.60
Conjugated syringic acid	0.90	0.30
Conjugated caffeic acid	0.50	0.30
Conjugated dimers	1.90	0.30
Total ferulic acid	76.40	6.40
Total vanillic acid	1.50	0.60
Total ρ-coumaric acid	4.50	1.70
Total syringic acid	16.60	6.90
Total caffeic acid	1.00	1.60
Total bound	72.60	6.60
Total free	18.50	6.60
Total conjugated	8.90	2.90
Total dimers	16.20	2.00

To calculate the mean and standard deviation values shown in the table we used all the lines and years. sd, standard deviation; dimers, dimers of phenolic compound; “total” is the sum of bound, free and conjugated phenolics. Percentages are calculate using absorbance data.

SD: standard deviation en %.

#### 3.2.1. Exploratory multivariate analysis (MANOVA and PC) of phenolic compounds content

We carried out a multivariate analyze to explore the whole data set. We fitted two models because the factor “Line” did not have repetitions inside of each year. Therefore, the Model 1 was: “variable ~ Line + Year”. The analysis of variance table showed that “Line” and “Year” had a highly significant effect on the data set (Table S1; Model 1). The Model 2 was fitted with main factors Year, Substitutions, translocation 1RS/1BL, Cytoplasm and the two interactions between them (‘variable ~ Year + 1RS/1BL + Substitutions+ Cytoplasm + Year × 1RS/1BL + Year × Substitutions + Year × Cytoplasm + 1RS/1BL × Substitutions + 1RS/1BL × Cytoplasm + Substitutions × Cytoplasm’). The main factors Year, 1RS/1BL and Substitutions had a significant effect on the data set (Table S1; Model 2). The interactions Year × Substitutions and 1RS/1BL × Substitutions also showed a significant effect. These results were used to simplify the models for the univariate analysis of each variable.

**Figure 2:**
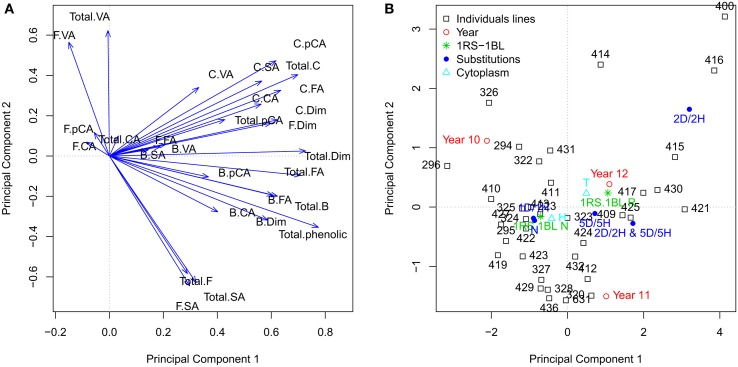
**Principal components analysis**. **(A)** The quantitative variables are represented. **(B)** The individuals lines and the centroids of levels factors “Year,” “Sustitutions,” “1RS/RL,” and “Cytoplasm” are represented.x “B.FA,” “F.FA,” “C.FA,” “Total.FA” are bound, free, conjugated and total ferulic acid; “B.VA,” “F.VA,” “C.VA,” “Total.VA” are bound, free, conjugated and total vanillic acid; “B.pCA,” “F.pCA,” “C.pCA,” “Total.pCA” are bound, free, conjugated and total *p*-coumaric acid; “B.CA,” “F.CA,” “C.CA,” “Total.CA” are bound, free, conjugated and total caffeic acid; “B.SA,” “F.SA,” “C.SA,” “Total.SA” are bound, free, conjugated and total syringic acid; “Total.B,” total bound phenolic; “Total.F,” total free phenolic; “Total.C,” total conjugated phenolic; “Total.phenolic,” total phenolic; “Total.Dim,” total dimer phenolic.

Principal component analysis (PC), based on correlation matrix, was performed using the different phenolic compounds contents and the factors with significant effects. According to the Kaiser–Harris criterion (Kaiser, 1974) we selected eight components with eigenvalues greater than 1. We used the principal component analysis as an exploratory analysis and not as a tool to reduce the number of variables. For this reason, we plotted the variables and factors regarding the principal component one and two, which accumulate the higher variability. Most of the variables are correlated with principal component 1 (PC1) being positive for all of them (Figure 2A). The PC2 showed a high correlation with the amounts of free vallinic acid, total vanillic acid, total free phenolic, free and total syringic acid. In this case, the amount of free and total syringic acid had a negative correlation with free vallinic acid, total vanillic acid and total free phenolics. The amount of free ρ-coumaric acid, free caffeic acid, bound syringic acid, free ferulic acid, bound vallinic acid and bound syringic acid showed a low correlation with PC1 and PC2.

The factors are represented on the PC1 and PC2 (Figure 2B). The factor “Year” had all its levels spread in the four different quadrants. The year 2011 and 2012 showed some variation along PC2; the year 2010 showed differences with respect to the year 2012 along of the PC1 and with the 2011 in both (PC1 and PC2). The “Substitutions” factor presented a wide dispersion of their levels. Disomic substitution DS2D(2H^ch^) and DS5D(5H^ch^) showed the higher differences regarding to the “no substitution” level, mainly along PC1. Disomic substitution DS2D(2H^ch^) showed the greatest differences regarding to the level “no substitutions” along both PC1 and PC2. The two levels of the factor “1RS/1BL” translocation showed difference in PC1. The “Line” factor showed a wide dispersion, although there is some accumulation in quadrant 3 (−, −), where the control levels of factors “Substitutions” and “1RS/1BL” are found.

#### 3.2.2. Univariate analysis of phenolic compounds content

We carried out a univariate analysis of variance to check which variables were affected by the factors studied. We fitted two models for the same reason that in the multivariate analysis of variance. Moreover, the MANOVA Model 1 and 2 showed factors with significant effects, consequently, we conducted univariate analysis of each of the variables using both models. In MANOVA Model 1 all factors, “Line” and “Year,” were significant, for that reason we use the same model (‘variable ~Line + Year’). In this analysis the factor “Line” had a significant effect (with a *p*-value <0.05) for all variables except conjugated FA, total VA, bound pCA, total CA and total conjugated (Table 3). The percentage of variance explained by the factor “Line” range between 57.6% and 28.2%. The factor “Year” had a significant effect on all variables except free FA, conjugated SA and free dimers. The percentage of variance explained by the factor “Year” range between 41.2% and 7.71%.

**Table 3:** **Table with the percentage of variance explained by each factors, derived from the ANOVA table of **Model 1** (‘variable ~ Line + Year’)**.

**Model 1**	**SS percentage**		
	**Line**	**Year**	**Residuals**
	**(Df = 36)**	**(Df = 2)**	**(Df = 72)**
Total ferulic acid	33.51^**^	37.21^*^	29.28
Conjugated ferulic acid	29.70	25.73^*^	44.57
Free ferulic acid	56.25^*^	0.04	43.71
Bound ferulic acid	36.02^**^	32.36^*^	31.63
Total vanillic acid	30.78	25.47^*^	43.74
Conjugated vanillic acid	42.07^*^	7.71^**^	50.22
Totalρ-coumaric acid	31.11^*^	35.29^*^	33.60
Conjugatedρ-coumaric acid	40.79^*^	33.34^*^	25.87
Free ρ-coumaric acid	53.76^*^	10.61^*^	35.62
Bound ρ-coumaric acid	28.44.	34.06^*^	37.51
Total syringic acid	39.96^*^	35.68^*^	24.36
Conjugated syringic acid	49.18^**^	1.21	49.61
Free syringic acid	40.25^*^	35.32^*^	24.43
Total caffeic acid	36.76.	12.51^*^	50.73
Conjugted caffeic acid	45.91^**^	8.41^**^	45.67
Total bound	33.49^**^	33.82^*^	32.70
Total free	37.34^*^	35.35^*^	27.31
Total conjugated	34.06.	21.59^*^	44.34
Total phenolic acids	28.21^*^	41.25^*^	30.54
Total dimers	56.69^*^	8.22^*^	35.10
Conjugated dimers	39.29^*^	15.05^*^	45.66
Free dimers	57.64^*^	2.04	40.32
Bound dimers	46.40^*^	17.35^*^	36.25

SS, sum of the squared; Df, degree of freedom.

Significant codes: 0 “***,” 0.001 “

** ,” 0.01 “

* ,” 0.05 “.,” 0.1 “ ”.

**Figure 3:**
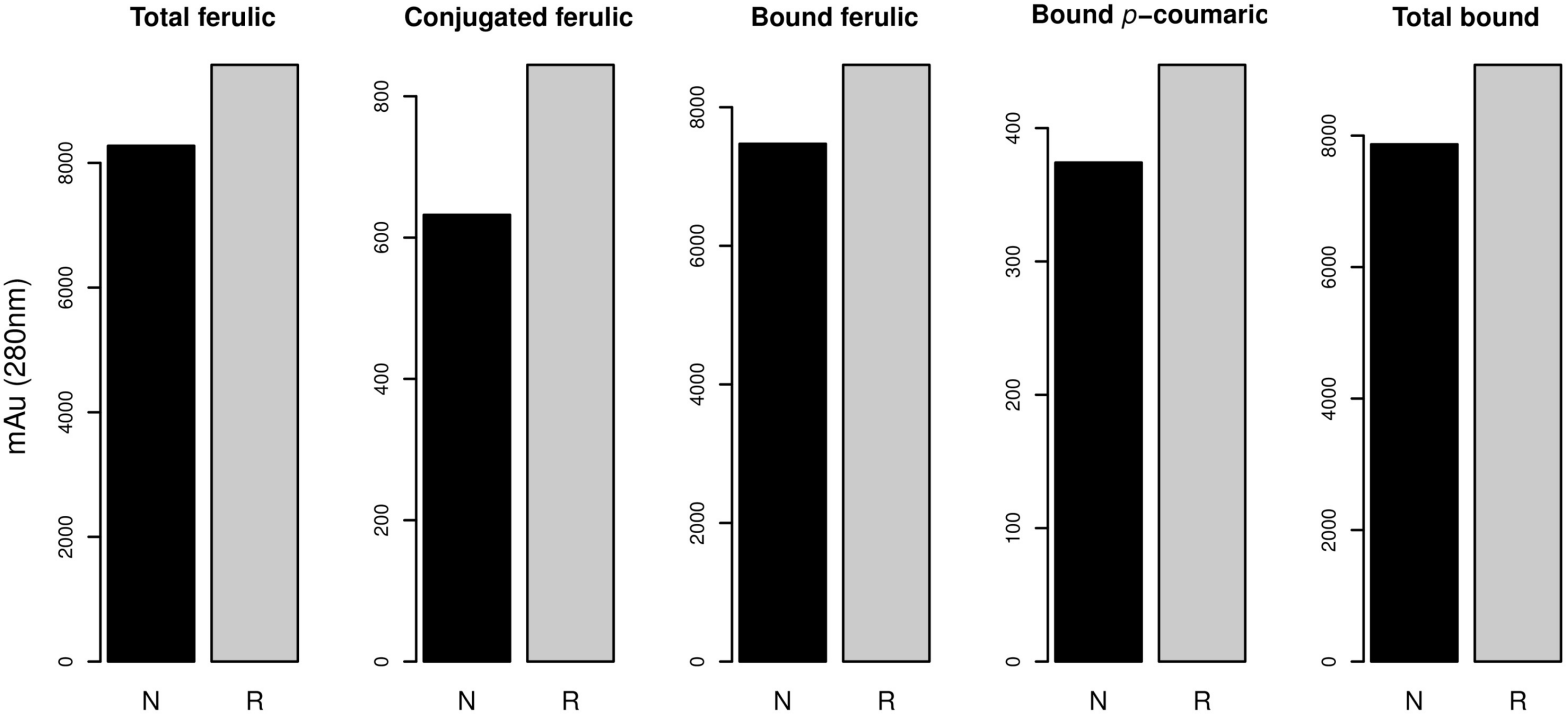
**Content of phenolic compounds with a significant effect of the translocation 1RS/RL (*p*-value < 0.05; Table 4)**. N, no translocation; R, translocation 1RS/RL.

From the results of MANOVA Model 2, we simplified the model by eliminating terms that were not significant. Therefore, we used the following Model 2 in the univariate analysis: “variable ~Year + Substitutions + 1RS/1BL + Year × Substitutions + Substitutions × 1RS/BL”. In this model the factor “Year” showed behavior almost identical to that shown in Model 1, showing even a very similar explained variance (Table 4). The factor “Substitution” showed a significant effect for all variables except bound pCA, total SA, free SA, total bound and total free. The variance explained by the factor “Substitution” ranged from 5.8% to 32.6% of the total variance. The factor “1RS/1BL” had a significant effect on total FA, conjugated FA, bound FA, bound pCA and total bound. The percentage of variance explained by this factor ranged from 2.2% to 3.6%. Interaction “Year × Substitutions” had significant effects on the variables conjugated FA, total conjugated and conjugated dimers, with percentages of explained variances from 15.8% to 18.7%. The last terms of Model 2, the interaction of “Substitution × 1RS/1BL” showed a significant effect for the variables total FA, bound FA, conjugated VA, total pCA, conjugated pCA, free pCA, conjugated SA and conjugated CA. The percentages of variance explained by this interaction are between 3.3% and 10.0%. The factor “1RS/1BL” as shown in Figure 3 increased the FA content in general,(soluble-conjugate, bound and total ferulic), bound pCA and total bound phenolics.

**Table 4:** **Table with the percentage of variance explained by each factors, derived from the ANOVA table of *Model 2* (‘variable ~ Year + Substitutions + 1RS/1BL + Year × Substitutions + Substitutions × 1RS/BL’)**.

**Model 2**	**SS percentage**					
	**Year (Df = 2)**	**Substitutions (Df = 4)**	**1RS/1BL (Df = 1)**	**Year × Subs. (Df =8)**	**Subs. × 1RS/1BL (Df = 2)**	**Residuals (Df = 90)**
Total ferulic acid	38.85^*^	6.92^*^	3.02^*^	1.68	3.29^*^	46.24
Conjugated ferulic acid	25.54^*^	12.30^*^	2.18^*^	18.70^*^	0.31	40.97
Free ferulic acid	0.23	14.49^**^	2.73.	3.41	3.24	75.90
Bound ferulic acid	33.41^*^	5.90^*^	3.02^*^	1.43	4.04^*^	52.21
Total vanillic acid	27.59^*^	6.51^*^	0.19	7.94	1.90	55.88
Conjugated vanillic acid	8.10^**^	10.63^**^	0.82	6.49	10.03^**^	63.93
Total ρ-coumaric acid	33.58^*^	6.43^*^	2.11.	6.07	3.66^*^	48.15
Conjugated ρ-coumaric acid	37.39^*^	14.79^*^	0.33	2.29	5.74^**^	39.46
Free ρ-coumaric acid	10.83^**^	10.12^*^	0.14	4.33	6.93^*^	67.65
Bound ρ-coumaric acid	32.70^*^	3.48	2.88^*^	1.51	2.17	57.26
Total syringic acid	35.58^*^	1.27	0.07	2.87	0.27	59.95
Conjugated syringic acid	1.01	22.86^*^	0.25	3.55	8.63^**^	63.69
Free syringic acid	34.96^*^	1.04	0.04	3.08	0.30	60.58
Total caffeic acid	12.53^*^	10.36^*^	0.06	2.37	2.52	72.16
Conjugted caffeic acid	8.82^**^	17.67^*^	0.02	4.28	8.45^**^	60.76
Total bound	34.88^*^	4.91.	3.62^*^	1.17	3.15	52.27
Total free	34.63^*^	3.30	0.25	2.59	0.59	58.63
Total conjugated	21.78^*^	16.05^*^	1.18	15.83^*^	1.04	44.12
Total phenolic acids	42.66^*^	5.79^*^	1.48.	0.83	1.81	47.43
Total dimers	8.96^*^	31.34^*^	0.23	3.28	3.32.	52.87
Conjugated dimers	15.70^*^	9.78^**^	2.21.	16.30^**^	0.43	55.58
Free dimers	2.17	32.64^*^	0.00	4.35	2.57	58.26
Bound dimers	18.91^*^	17.49^*^	0.36	3.31	3.41.	56.52

SS, sum of the squared; Df, degree of freedom.

Significant codes: 0 “^***^,” 0.001 “

** ,” 0.01 “

* ,” 0.05 “.,” 0.1 “ ”.

**Figure 4:**
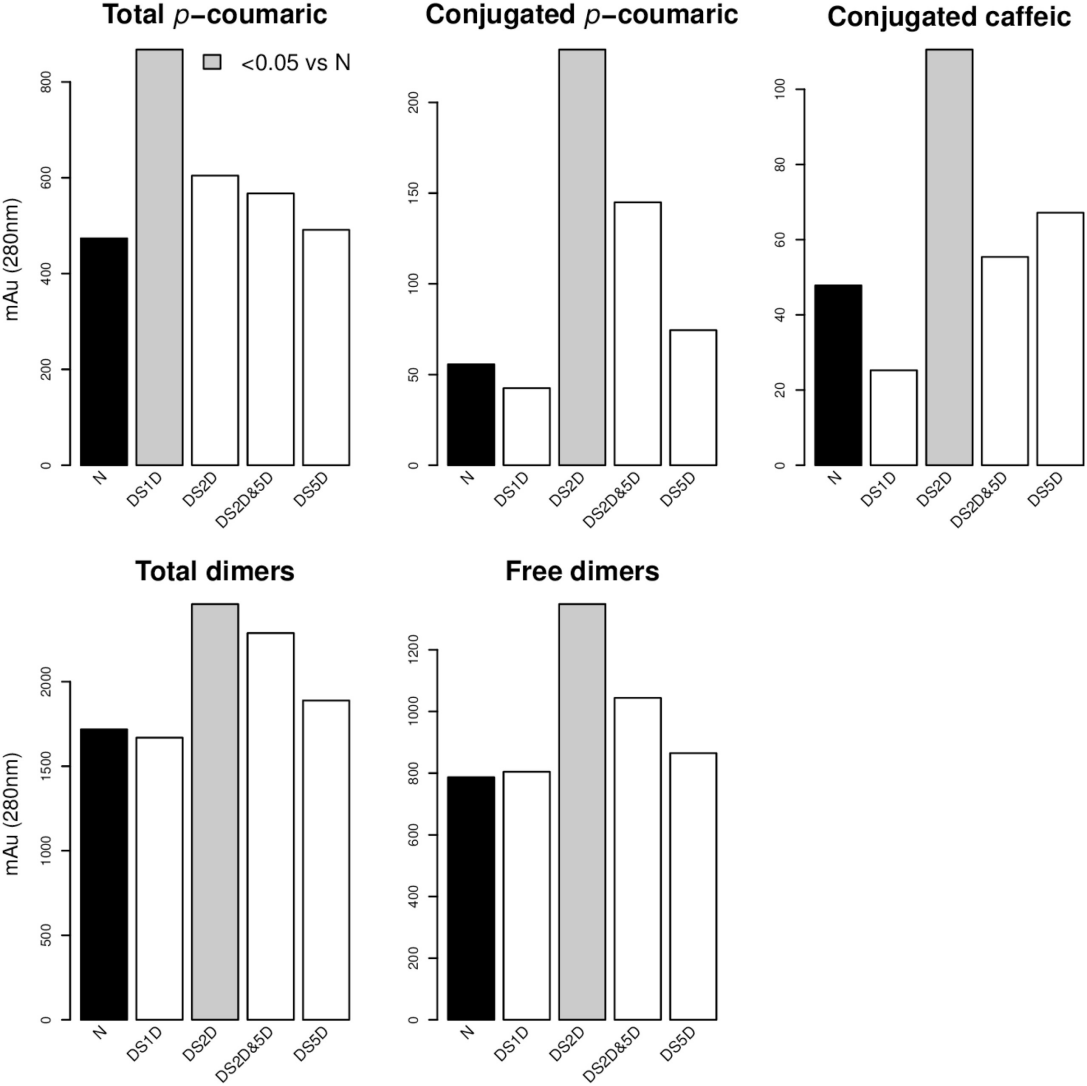
**Phenolic acid content and multiple-comparison results of the different substitution lines**. Gray bars are significantly different (*p*-value < 0.05) compared to the no substitution (N). Comparison of means (Tukey contrast) between levels of factor “Substitutions” took place only when the ANOVA table of Model 2 (Table 4) showed a significant effect of this factor. Figure shows only those compounds where any substitution level showed differences with respect to no substitution level (N).

For the factor “Substitution” we carried out a comparison of means between the different levels (Figure 4). Chromosome substitution DS1D(1H^ch^) caused an increase of total pCA. The substitution DS2D(2H^ch^) was the substitution with the higher effect on phenolic compounds. This substitution increased total pCA, conjugated pCA, conjugated CA, total dimers and free dimers. In fact, three of the four lines with the highest contents of phenolic compounds had the substitution DS2D(2H^ch^)(lines 400, 415, and 416).

## 4. Discussion

Phenolic compounds of whole grain are an important trait because they have several human health benefits. Wild cereal species are an important source of variability that can be used by plant breeders to produce healthier wheat varieties. The amphiploid tritrodeum is an excellent way to access the variability present in the wild species *H. chilense*. In this paper we characterize the phenolic acid composition of a collection of tritordeums, and we assess the effect of growing season, the barley or wheat cytoplasm, chromosomal substitutions and the widespread chromosomal translocation 1RS/1BL.

Until now, there was little information on the composition of phenolic acids, the main compounds with antioxidant activity, of the amphiploid tritordeum. Ferulic acid is the predominant phenolic acid found in whole grains (Krygier et al., 1982; Mattila et al., 2005) and whole wheat (Moore et al., 2005; Li et al., 2008). These authors have reported ferulic acid percentages to the total of phenolic compounds in the range of 72–85%, in the case of Li et al. (2008) and 83.5–89.5% in the case of Moore et al. (2005). The percentages of ferulic acid with respect to total phenolics in our tritordeum collection (on average 76.38%) are similar to those found in wheat. In our study, 90.2% of total ferulic acid is found in the fraction of bound phenolic acids. These values agree with those reported in wheat, since several authors have shown percentages of ferulic acid in the bound fraction from 89% to 97% (Adom et al., 2003; Moore et al., 2005; Li et al., 2008).

Other phenolic acids were also found in significant quantities in whole wheat. Okarter et al. (2010) have reported that the second most abundant phenolic acid found in whole wheat is ρ-coumaric acid. In this study, the second more abundant phenolic compound is the syringic acid and not the ρ-coumaric acid, which was found in smaller amounts than syringic acid (16.6% of total phenolic acids is syringic acid and only the 4.52% of total phenolics is ρ-coumaric acid). The higher ratio of syringic acid in grain has been also reported by Li et al. (2008) in spring and spelt wheat and some varieties of soft wheat (Moore et al., 2005).

Syringic and vanillic acids are hydroxybenzoic acids, and ferulic acid, ρ-coumaric acid and caffeic are hydroxyninnamic. The hydroxyninnamic and hydroxybenzoic acids belong to different biosynthetic pathways; hydroxybenzoic acids belong to aminobenzoate pathway, and the hydroxyninnamic acids to the phenylpropanoid biosynthesis pathway. Figure 2A shows a high correlation of the total hydroxybenzoic acids (total vanillic acid syringic total acid) with PC2, unlike most hydroxyninnamic acids (ferulic acid, caffeic acid and ρ-coumaric acids) are correlated with PC1. This may be because the hydroxybenzoic acids and hydroxyninnamic acids belong to different pathway and these pathways are not strongly correlated.

Several authors have shown high and positive correlations between total free, conjugated and bound phenolic acids (Verma et al., 2008; Fernandez-Orozco et al., 2010). In our study there is a strong correlation between total conjugated and united phenolic. On the contrary, the total free phenolics is less correlated with the above (total bound and conjugated phenolics) in contrast to that reported by Fernandez-Orozco et al. (2010). Syringic acid is the compound with mayor contribution to the total free phenolics, while ferulic acid is the main phenolic compound of bound and conjugated phenolics. The low correlation observed between the free phenolics with conjugated and bound phenolics may be due to that the ferulic acid and syringic acid belong to different biosynthetic pathways, as we have described above.

The environmental effect (the growing season and location) has been proposed as the main factor that determines phenolic acid content. Environmental factors such as solar radiation, temperature, and irrigation, affect the content of phenolic acids in wheat but some phenolics fractions are more susceptible to the effect of environment than others (Moore et al., 2006; Fernandez-Orozco et al., 2010). Mpofu et al. (2006) has shown that the effect of the environment is greater than the effect of genotype on hard and spring wheat. Our study corroborates the fact that the environment has a strong effect on the content of phenolic acids, as shows the effect of the factor “Year” in our analysis. Conversely, our study shows that although the effect of the environment is high the genotype has a greater effect in many of the analyzed phenolics fractions. In fact, the factor “Year” only had a greater impact than genotype on the total ferulic acid, total ρ-coumeric, bound ρ-coumeric and total phenolic. Our study shows a greater effect of factor “genotype” that has not been previously shown in wheat. This may be due to the higher genetic variability of tritordeum in comparison to wheat. This variability gives amphiploid tritrordeum a high value as source of genetic variability to improve the antioxidant content of wheat, or as a cereal food crop in its own right.

As shown in Figure 4, the chromosome substitution DS2D(2H^ch^) increases the content of hydroxyninnamic acids (caffeic acid and ρ-coumaric acids). We also observed an effect on the phenolic acid content of the substitution DS1D(1H^ch^), since it increases the total dimers. This suggest that either the absence of chromosome 2H^ch^ or the introgression of chromosome 2D leads to increase the content of phenolic compounds. The widely used translocation 1RS/1BL increases the content of total, conjugated and bound ferulic acid, bound ρ-coumaric acid and total bound phenolics in tritordeum. This is the first work where the phenolic acid content is associated with the substitution of chromosome DS1D(1H^ch^) and DS2D(2H^ch^), and the translocation 1RS/1BL in tritordeum. Therefore, on chromosome 1 and 2 may be located genes involved in hydroxyninnamic acids biosynthesis and/or accumulation.

In conclusion, the high variability of phenolic compounds present in tritordeum confers this a high potential for improving the quality and quantity of dietary antioxidants in cereals. However, in this connection, some limitations are worth noting. The transfer of traits from wild relative species to wheat can be achieved by uncontrolled translocations which may also introduce non-desired traits, or by the identification of genes involved in the trait and its use for wheat transformation. Thus, although tritordeum is very interesting source of genetic variability for phenolic acid content more in depth understanding of the genes that determine the content of phenolic compounds is needed for directed improvement of antioxidants in tritordeum or in related cereals.

## Funding

This work was supported by the Spanish Comisión Interministerial de Ciencia y Tecnología (AGL2011-22596). FP is supported by “Ramón y Cajal” research contract from the MINECO (RYC-2010-07345).

### Conflict of interest statement

The authors declare that the research was conducted in the absence of any commercial or financial relationships that could be construed as a potential conflict of interest.

## Supplementary material

Table S1 **ANOVAs tables from the MANOVA analysis of the Model 1 (‘variable ~ Line + Year’) and Model 2 (‘variable ~ Year + 1RS/1BL + Substitutions + Cytoplasm + Year × 1RS/1BL + Year × Substitutions + Year × Cytoplasm + 1RS/1BL × Substitutions + 1RS/1BL × Cytoplasm + Substitutions × Cytoplasm’)**.

Figure S1 **RP-HPLC chromatograms of the pure phenolic compounds studied in this work**. For this analysis we used pure phenolics from SIGMA-ALDRICH. Mix, a mix of the same amount of each phenolic acids. FA, ferulic acid; pCA, ρ-coumaric acid; CA, caffeic acid;VA, vanillic acid; SA, syringic acid.

Figure S2 **Spectra of the pure phenolic acids**.
