# Supplementary figures and images for: Phenolic content variability and its chromosome location in tritordeum
Source: Front Plant Sci. 2014 Jan 30;5:10. doi: 10.3389/fpls.2014.00010 (PMC3906567; doi:10.3389/fpls.2014.00010)

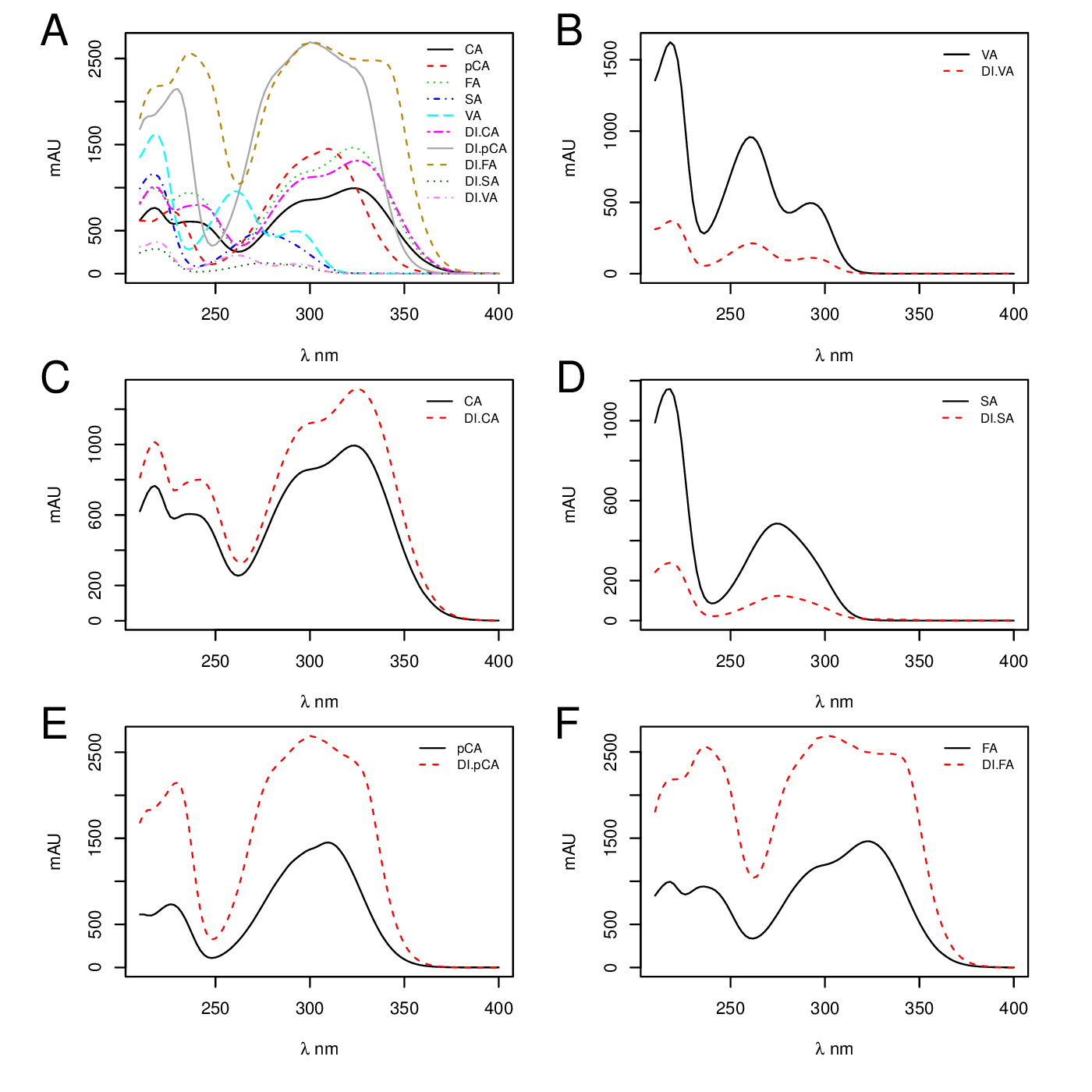

Supplement: Table S1 — ANOVAs tables from the MANOVA analysis of the Model 1 (‘variable ~ Line + Year’) and Model 2 (‘variable ~ Year + 1RS/1BL + Substitutions + Cytoplasm + Year × 1RS/1BL + Year × Substitutions + Year × Cytoplasm + 1RS/1BL × Substitutions + 1RS/1BL × Cytoplasm + Substitutions × Cytoplasm’). [file Presentation1.ZIP › 73889_Pistón_Suppl Figure_2.TIFF]
